# Book Review

**DOI:** 10.1038/sj.bjc.6690732

**Published:** 1999-10

**Authors:** Gareth Morgan

**Affiliations:** Division of Clinical Sciences Algernon Firth Building University of Leeds Leeds LS2 9JT


*British Journal of Cancer* (1999) **81**(3), 564
© 1999 Cancer Research Campaign
Article no. bjoc.1999.0732

### Book Review

### The Clinical Practice of Stem Cell Transplantation

Barrett and Treleaven, pp. 950, ISBN 1 8990 6670 5.
John Harrison, Oxford, UK; £175.

This is a well laid out and presented book that covers all aspects of the clinical practice of stem cell transplantation. The first section summarizes the evidence for the clinical use of high-dose chemotherapy procedures and is broken down into chapters on individual diseases. This is generally well done and, in most cases, all of the relevant literature is summarized. However, there is a tendency to dwell on numerous case series rather than to concentrate on the data available from randomized studies. Overall, this is a good section which summarizes the available literature, though often in an idiosyncratic fashion, and also provides a repository of useful references.

The second and third sections describe the practical aspects of the chemotherapeutic procedures and the supportive care of patients undergoing them. In comparison to the first, these two sections are pitched at a different level. The practical aspects of stem cell transplantation are now all too obvious to people working in the field and this section seems to be aimed at providing an introduction for people coming to the field for the first time. It does this well, but as a consequence tends to be a little superficial. The section on problems after transplantation is split into five sub-sections: graft-versus-host disease; rejection and relapse; infections; early complications; and late complications. This is a useful approach, but again, similar to the previous section, it has a tendency to provide introductory advice rather than in-depth discussion. This is perhaps not surprising as it would be difficult to discuss fully the infective complications after transplantation in any other fashion in a book of this size.

The fourth and final section is a hotch potch of different aspects of transplantation. The organization of the book is good and it is well illustrated, which helps to make it easy to read and easy to find useful information. It is generally well referenced and provides easy access to relevant literature. It may have benefited from a reduction in size and omission of some chapters.

The book is aimed at health care workers in the field of bone marrow transplantation, including doctors, nurses and pharmacy staff. Overall, the book provides an excellent overview of the practice of stem cell transplantation, but is large at nearly 1000 pages and is expensive at £175. It is, however, comprehensive and they have achieved their aim of providing an all encompassing text. The down side is that some of the supportive care sections tend to be superficial. I would have no difficulty in recommending it for haematologists, oncologists, nurses and other health care workers. As well as providing a reference text for transplant units, it also forms a good basis for tutorials and exam preparation. Inevitably, because of the time to publication, the book is less useful in its discussions on recent advances in stem cell transplantation and basic scientific issues are only superficially addressed.

*Gareth Morgan*
*Division of Clinical Sciences*
*Algernon Firth Building*
*University of Leeds*
*Leeds LS2 9JT*

